# Nondestructive detection of *Pleurotus geesteranus* strain degradation based on micro-hyperspectral imaging and machine learning

**DOI:** 10.3389/fpls.2023.1260625

**Published:** 2023-12-06

**Authors:** Xuan Wei, Shiyang Liu, Chuangyuan Xie, Wei Fang, Chanjuan Deng, Zhiqiang Wen, Dapeng Ye, Dengfei Jie

**Affiliations:** ^1^ College of Mechanical and Electrical Engineering, Fujian Agriculture and Forestry University, Fuzhou, Fujian, China; ^2^ College of Future Technology, Fujian Agriculture and Forestry University, Fuzhou, Fujian, China; ^3^ College of Life Science, Fujian Agriculture and Forestry University, Fuzhou, Fujian, China

**Keywords:** edible fungi, micro-hyperspectral imaging, phenotype, strain degradation, classification

## Abstract

In the production of edible fungi, the use of degraded strains in cultivation incurs significant economic losses. Based on micro-hyperspectral imaging and machine learning, this study proposes an early, nondestructive method for detecting different degradation degrees of *Pleurotus geesteranus* strains. In this study, an undegraded strain and three different degradation-level strains were used. During the mycelium growth, 600 micro-hyperspectral images were obtained. Based on the average transmittance spectra of the region of interest (ROI) in the range of 400-1000 nm and images at feature bands, feature spectra and images were extracted using the successive projections algorithm (SPA) and the deep residual network (ResNet50), respectively. Different feature input combinations were utilized to establish support vector machine (SVM) classification models. Based on the results, the spectra-input-based model performed better than the image-input-based model, and feature extraction improved the classification results for both models. The feature-fusion-based SPA+ResNet50-SVM model was the best; the accuracy rate of the test set was up to 90.8%, which was better than the accuracy rates of SPA-SVM (83.3%) and ResNet50-SVM (80.8%). This study proposes a nondestructive method to detect the degradation of *Pleurotus geesteranus* strains, which could further inspire new methods for the phenotypic identification of edible fungi.

## 1 Introduction

As strain quality directly affects the yield of edible fungi, it is important to prevent strain degradation and maintain strain stability during the preservation process. Strain degradation refers to the deterioration of the edible fungi population, which leads to decreases in yield, quality, resistance, and so on (Qiu et al., 2010; Yin et al., 2017). In the production of edible fungi, the use of degraded strains in cultivation will lead to significant economic losses. Therefore, it is important to rapidly identify the degraded strains to avoid using them to produce edible fungi. Currently, the detection methods mainly use chemical or biological technology to analyze the health condition of strains (Magae et al., 2005; Sun et al., 2017; Chen et al., 2019; Xin et al., 2019; Zhao et al., 2022). Not only are these methods time-consuming and complex, they may even lack accuracy. Therefore, it is necessary to explore an alternative method to quickly and accurately identify the early stages of strain degradation.

With the advancements in imaging and spectroscopy, hyperspectral imaging (HSI) has been used to detect the contents of edible fungi, such as moisture and polysaccharides. Gaston et al. (2010) used hyperspectral imaging to determine the activities of the polyphenol oxidase enzyme in the caps of damaged *Agaricus bisporus* in order to rapidly identify mushrooms with a higher possibility of enzymatic browning. Xiao et al. (2020) evaluated the soluble solids in *Agaricus bisporu*s slices based on hyperspectral images during ultrasonic-assisted osmotic dehydration. In our previous study, HSI and support vector machines (SVM) were used to detect *Agaricus bisporus* diseases rapidly. In these studies, HSI demonstrated good performance in the field of edible fungi. However, these studies were focused on the fruiting bodies rather than strains, which play an essential role in edible fungi cultivation (Sun et al., 2017). Therefore, methods to detect the health of mycelia at the microscale need to be explored.

Micro-hyperspectral imaging (MHSI) combines micro-optical imaging technology and spectral analysis technology. It has been widely used in medical diagnosis (Hu et al., 2019; Hu et al., 2020; Lv et al., 2021), food safety detection (Zhu et al., 2020; Huang et al., 2021; Jiao et al., 2021), and other fields. Based on the transmittance MHSI technique, Xu et al. (2020) used the support vector machine (SVM) model to distinguish two microalgae with an accuracy and specificity of 94.4% and 97.2%, respectively. Furthermore, they predicted the growth stage of *Phaeocystis* with an accuracy of 98.1% by the random forest (RF) method. Additionally, Ortega et al. (2018) created spectral libraries of tissues from micro-hyperspectral images at 400-1000 nm and processed them using three different supervised classification algorithms. The results showed the ability of MSHI to accurately discriminate between normal and tumor tissue. Wu et al. (2022) obtained the micro-hyperspectral images of tomato leaves to detect POD activity under different salt stresses; the *R^2^* and RMSEP of the partial least squares regression (PLSR) model were 0.66 and 18.94 U/g-min, respectively. These studies demonstrated that MHSI had a good performance at the microscale.

Microscopic technology can observe the morphological structure and internal characteristics of strains. Therefore, in combination with the advantages of HIS, it is considered that the MHSI can be used to detect and analyze the degradation of strains. This study proposes to use MHSI and machine learning to discriminate the degree of degradation in the *Pleurotus geesteranus* strains. The study findings are expected to provide new insights and continuous monitoring methods for the early identification of the degradation of edible fungi strains.

## 2 Materials and methods

### 2.1 Experimental materials

**Figure 1:**
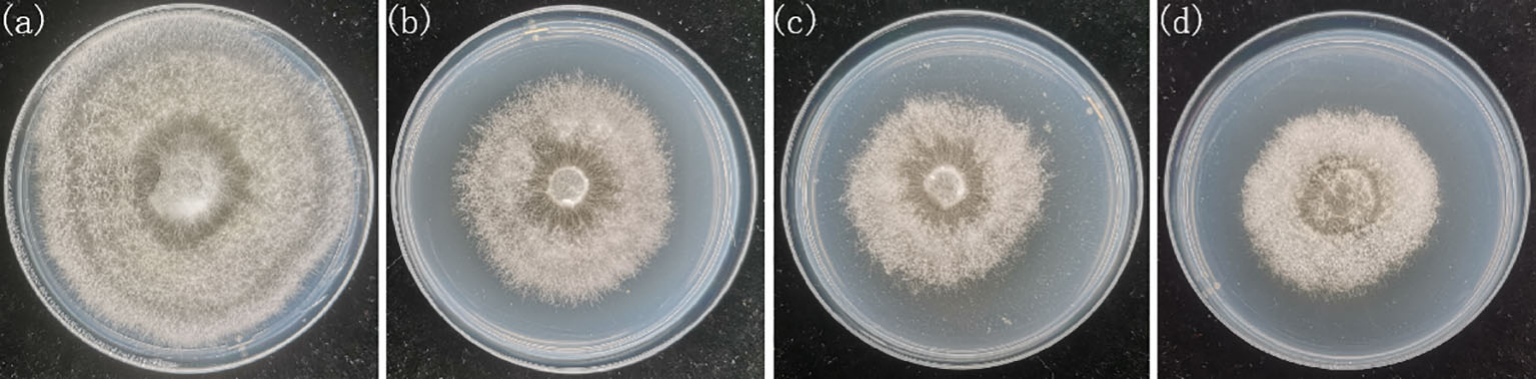
RGB pictures of undegraded and different degraded *Pleurotus geesteranus* strains **(A)** strain Xiu57-0, **(B)** strain Xiu57-1; **(C)** strain Xiu57-2; **(D)** strain Xiu57-3.

In this study, the slightly degraded strain of *Pleurotus geesteranus* (Xiu 57-1) was subcultured into eighth and fifteenth generation, respectively. And the non-degraded strain (Xiu 57) was used for comparison. The strains were obtained from the Mycological Research Center of Fujian Agriculture and Forestry University. The strains were inoculated into the center of culture dishes containing potato dextrose agar (PDA) medium (Ye et al., 2022) and placed in a 25°C room for 10 days to activate them (**Figure 1**). The growth rate and enzyme activity of the four types of strains were tested, and the degradation degrees of these four strains were then ranked. The activity of laccase, carboxymethylcellulase (CMC) and xylanases were determined using test kits (Suzhou Comin Biotechnology Co., Ltd., Suzhou, Fujian, China), respectively. The samples were each duplicated three times. The samples were labeled Xiu57-1, Xiu57-2, and Xiu57-3 from low to high degradation degrees, and the non-degraded strain was labeled Xiu57-0. In the experiment, a piece of flora with a diameter of 5 mm at the end of the activated mycelium was inoculated into the center of a new 90 mm culture dish containing PDA. Then, these culture dishes were placed upside down in a 25°C room, and images were collected after 5 days of culture. In total, we collected 200 samples, comprising 50 samples for each degradation degree.

### 2.2 Data acquisition

#### 2.2.1 Micro hyperspectral image acquisition

**Figure 2:**
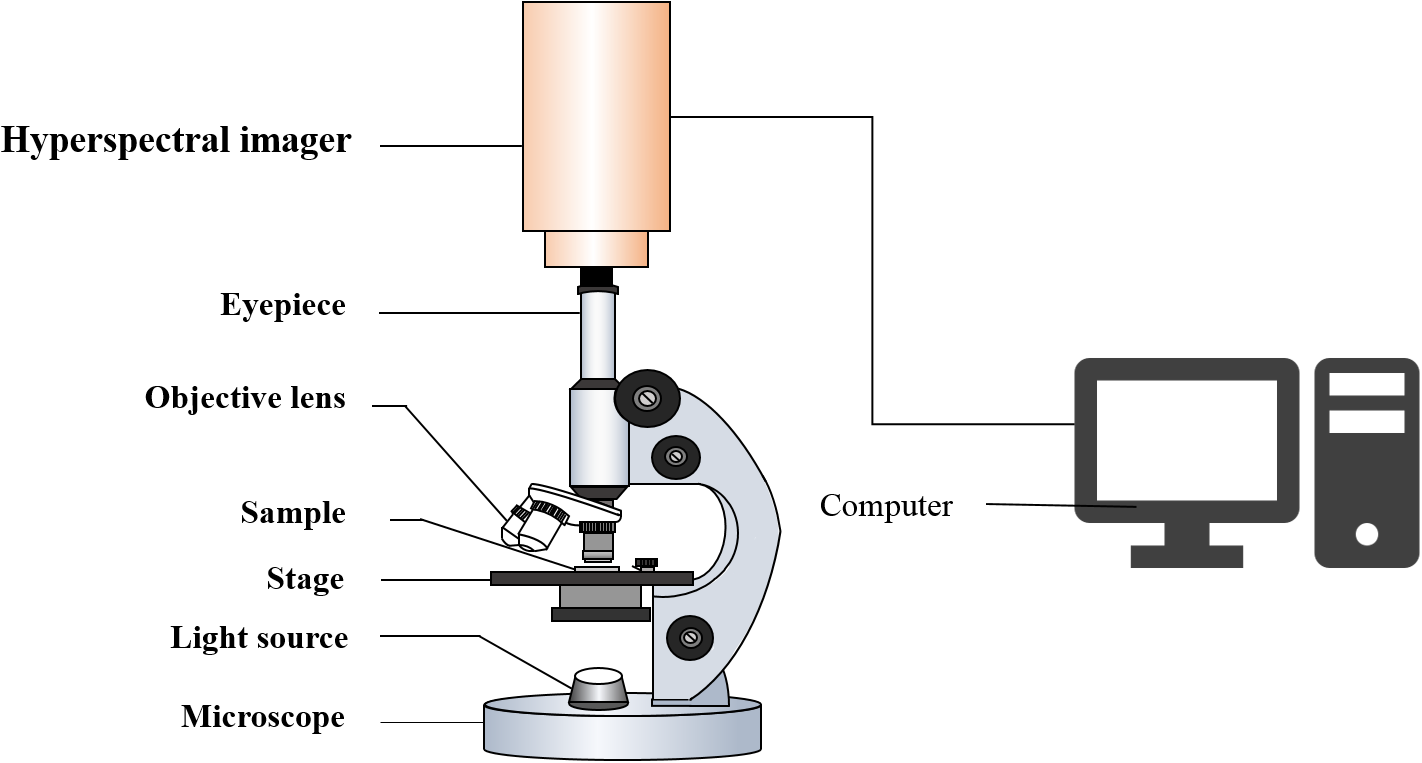
Micro-hyperspectral imaging system.

The MHSI system used in this study consisted of an optical microscope (BX53, Olympus, Japan), built-in push-broom portable hyper spectrometer (GaiaField Pro-V10E, Sichuan Dualix Spectral Imaging Technology Co., Ltd., China), light source, and computer (**Figure 2**). The hyperspectral images of the *Pleurotus geesteranus* strains were magnified 100 times (eyepiece 10X, objective lens 10X). The spectra were in the wavelength range of 401-1046 nm and contained 360 wavelength variables (sampling interval was 1.79 nm) with a spectral resolution of 2.8 nm and an image resolution of 960 × 861 dpi. The camera exposure time was set to 15 ms, and the collection speed was 0.06 cm/s. Three positions in each culture dish were randomly selected to collect data for each strain.

#### 2.2.2 Correction of micro-hyperspectral images

To reduce the noise caused by the light intensity and dark current, black-and-white correction was conducted after acquiring the micro-hyperspectral image data. In this study, the micro-hyperspectral image of the uninoculated PDA medium was collected as the whiteboard data, and the cap was used to block the light to collect the dark background. Then, it was corrected according to Eq. (1):


(1) $$R=\;\frac{I_{raw}-I_{dark}}{I_{ref}-I_{dark}\;},$$

Where: $I_{raw}$ represents the sample images, $I_{dark}$ is the dark background data, and $I_{ref}$ is the whiteboard data.

### 2.3 Data pretreatment

#### 2.3.1 Extraction of region of interest

The region of interest (ROI) was manually created in the mycelial region of the micro hyperspectral image using the New Region of Interest tool in ENVI5.3 (Exelis Visual Information Solutions, Inc., USA). The average spectral transmittance was calculated for pixels in the region.

#### 2.3.2 Spectral pretreatment

In order to further reduce the noise or scattering, spectral preprocessing was performed using different methods: the Savitzky-Golay smoothing (SG smoothing), multivariate scatter-correction (MSC), and standard normalized variate (SNV).

The SG smoothing uses the method of local polynomial least square fitting to replace each value of the signal sequence with a new value (Jahani et al., 2018). MSC uses the average of the spectral data as the “ideal spectrum”. It corrects the baseline shift and offset of the spectral data through the ideal spectrum, so as to effectively eliminate spectral differences due to different scattering levels (He et al., 2022). SNV normalizes the original spectral data according to the mean value and standard deviation of spectra, and each spectrum is corrected separately (Guezenoc et al., 2019; Mishra et al., 2020).

### 2.4 Feature selection

Dimension reduction is necessary to improve the accuracy of hyperspectral images in classification applications. Depending on the feature in spectra and spatial dimensions, dimension reduction methods can be band selection and feature extraction, respectively.

#### 2.4.1 Feature band selection

Successive projection algorithm (SPA) is a variable selection method for multivariate calibration. It uses the simplest operations in the vector space and aims to improve the conditions of multiple linear regression by minimizing the collinearity effect in the calibration dataset (Wei et al., 2020). Let the spectra set as *X_ca_*_l_, the initial wavebands set as *k(0)*, *N* set as the number of selected variables, the calculation step is as follows (Araújo et al., 2001; Sun et al., 2019):

Step 1: initialization: $n=1,\;x_{j}\;=\;j\;th\;column\;of\;X_{cal},\;j=1,\cdots ,\;J;$

Step 2: Let *S* is the set of unselected variables S= {j, 1≤j≤J, j∉{k(0),⋯,k(N−1)}} ;

Step 3: Calculate the projection of xj on the subspace orthogonal to $x_{k\left(n-1\right)}$ as


(2) $$Px_{j}=\;x_{j}-\left(x_{j}^{T}x_{k\left(n-1\right)}\right)x_{k\left(n-1\right)}{\left(x_{k\left(n-1\right)}^{T}x_{k\left(n-1\right)}\right)}^{-1}$$

Where *P* is the projection operator;

Step 4: Let $k\left(n\right)=arg\left(max\|Px_{j}\|,\;j\in S\right)$ ;

Step 5: Let n=n+1. If *n< N* go back to Step 1.

End: the selected wavelengths are {k(n); n =0,⋯,N−1} .

#### 2.4.2 Image feature extraction

In this study, a pre-trained convolutional neural network (CNN) model was used to extract image features. The principle was to update a feature set infinitely in backpropagation based on an initialized distribution. The two core elements of this process were the convolution kernel and the input image. For the input image, the convolution kernel slid over it and calculated the dot product between the input matrix and the convolution kernel at each spatial position. After the convolution process, each kernel was convolved with the input image to calculate a new feature map. Since the feature set was infinitely similar to the conceptual eigenvector in mathematics, the matrix was extracted by the mathematical method of eigenvectors. The specific formula is as follows:


(3) $$h^{k}=f\left(W^{k}*x+b^{k}\right),$$

Where, the deviation *b* and weight *W^k^* are shared parameters and *h^k^* is the feature mapping generated after the convolution calculation for subsequent convolution calculations.

In order to get more characteristics, the layer of convolution was selected more. However, the increase in network depth could elicit problems of saturation and rapid degradation of network training accuracy (Wang et al., 2019). To solve these issues, the Residual Network (ResNet), which added residual learning based on the deep convolutional neural network, was first proposed in 2015 (He et al., 2016).

**Figure 3:**
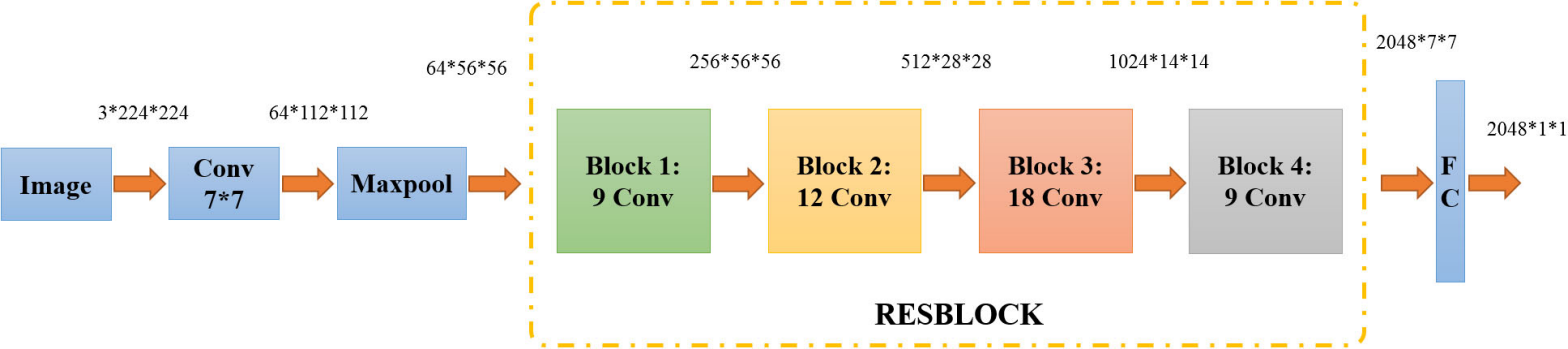
The structure of ResNet50.

As shown in **Figure 3**, the ResNet50 network is divided into seven parts, including 49 convolutional layers and one fully connected layer. At first, the network performed convolution, regularization, activation function, and max pooling on the input part, which did not contain residual blocks. Then, there were four residual blocks. In the next step, the full connection operation was performed to calculate the feature vector obtained by the model (Jie et al., 2021). Ultimately, image feature extraction was completed.

### 2.5 SVM classification model

SVM is a supervised machine learning method based on generalized linear classifiers. Its main goal is to find the best hyperplane for the classification of new data points (Sharma et al., 2017; Tiwari and Ojha, 2019). This is achieved by maximizing the margins between different classes and reducing the distance between the hyperplane focuses. To identify a suitable SVM, it is necessary to find *a_i_* and *b* by minimization, and then optimize and express them as follows (Tang et al., 2020):


$$\max\left(\sum_{i=1}^{N}\alpha_{i}-\frac{1}{2}\sum_{i=1}^{n}\sum_{i=1}^{n}y_{i}\alpha_{i}k\left(x_{i}x_{j}\right)y_{i}\alpha_{i}\right),\;\sum_{i=1}^{n}\alpha_{i}y_{i}=0,$$

(4) $$0\leq \alpha_{i}\leq C,\;i=1,\cdots ,n$$

where $k\left(x_{i},x_{j}\right)$ is the kernel function; C is the regularization parameter representing the error classification tolerance parameter.

In the input space, the training data will be projected into a higher dimensional feature space when the linear separation in the kernel function becomes easier. SVM utilizes different kernel functions in order to find a hyperplane that could divide the data into groups more efficiently. It has good classification performance when used for the minimum training set (Pitchai et al., 2023). Therefore, the SVM model needs an appropriate kernel function in order to correctly evaluate the hyperplane and reduce classification errors. In this study, the kernel function of SVM is defined by means of weighted summation. The specific algorithm formula is as follows:


(5) $$K\left(x_{i},x_{j}\right)=\mu K_{s}\left(x_{i}^{s},x_{j}^{s}\right)+\left(1-\mu \right)K_{w}\left(x_{i}^{w},x_{j}^{w}\right)$$

Wherein, μ is the weighting factor, 0 ≤ μ ≤ 1; $K_{S}$ is the spatial kernel function and $K_{w}$ is the spectral kernel function.

### 2.6 Evaluating indicator

In order to evaluate the classification effect of the model on samples, this study selected the accuracy of detection classification as an indicator to qualitatively evaluate the model. The accuracy rate is the ratio of the number of correctly classified samples to the total number of samples. A higher accuracy rate indicates a better classifier. The specific calculation formula is as follows (Jin et al., 2018):


(6) $$Accuracy=\frac{TP+TN}{TP+TN+FP+FN}$$

In the above formula,

*TP* (true positives): the positive class is judged to be the positive class.
*TN* (true negatives): the negative class is judged to be the negative class.
*FP* (false positives): the negative class is judged to be the positive class.
*FN* (false negatives): the positive class is judged to be the negative class.

## 3 Results

### 3.1 Degradation degree of the strains

The determined extracellular enzyme activities of the samples were shown in **Table 1**. It shows that there were significant difference of laccase CMC and xylanase activity among different groups(p<0.05). Compared to the non-degraded strain(Xiu 57), the degraded strains exhibited decreased enzyme activities. Furthermore, as subculture generation increased, all three enzymes activities of the strains significantly declined. In fact, these activities could decrease by more than half compared to those of the non-degraded strain.

**Table 1:** Three different enzyme activity values.

NO.	Extracellular enzyme activity(U/L)		
	Laccase	CMC	Xylanase
Xiu 57-0	166.74^a^	947.75^a^	1278.90^a^
Xiu 57-1	120.34^b^	686.60^b^	803.48^b^
Xiu 57-2	93.98^c^	479.90^c^	564.05^c^
Xiu 57-3	78.68^d^	350.18^d^	458.20^d^

a, b, c, and d are significant differences at the 0.05 level.

### 3.2 Spectra of *Pleurotus geesteranus* strain samples

**Figure 4:**
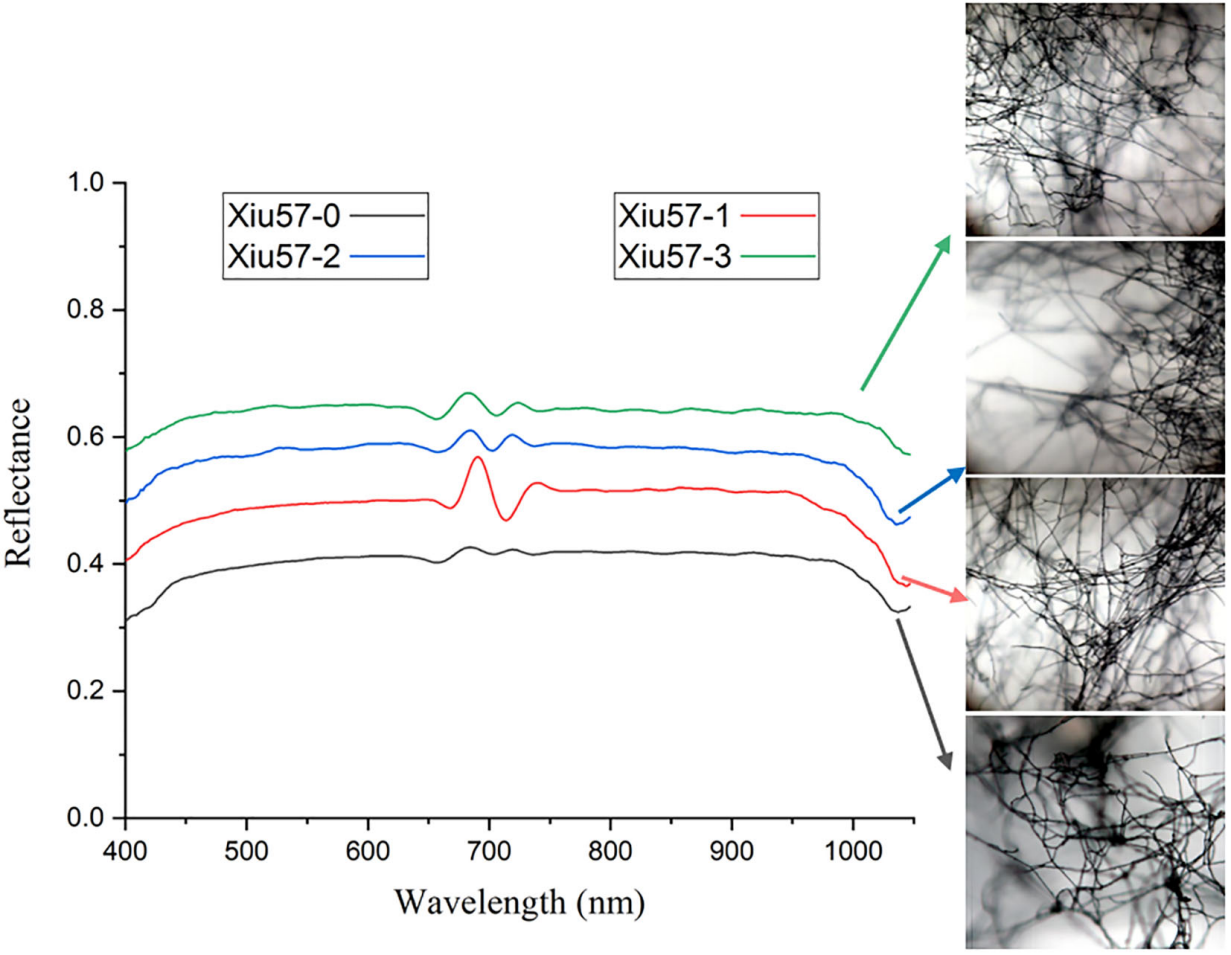
Micro-hyperspectral images and average spectral curves of undegraded and degraded strain of *Pleurotus geesteranus*.

In this study, a total of 600 micro-hyperspectral images of undegraded and three differently degraded strains of *Pleurotus geesteranus* were collected, including 150 images each of 57-0, 57-1, 57-2, and 57-3. Next, 600 average spectral data points were obtained from the ROI region of mycelium, and the curves are displayed in **Figure 4**. As shown in the figure, the four *Pleurotus geesteranus* strains had similar mycelial textures and hyperspectral curve trends. With increased degradation, the mycelia became more narrow and the transmittance slightly increased. Correspondingly, the mycelia of the undegraded strain of *Pleurotus geesteranus* were relatively stronger, and the transmittance curve was slightly lower than that of the degraded strain.

### 3.3 Pretreatment

**Figure 5:**
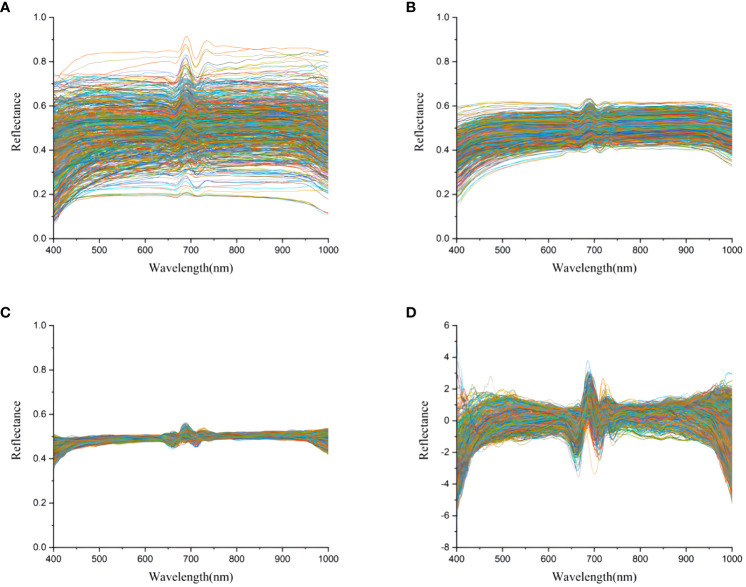
Transmittance curves of the mushroom strain of *Pleurotus geesteranus.* **(A)** original average spectral transmittance curve; **(B)** average spectral transmittance curve after SG; **(C)** average spectral transmittance curve after MSC; **(D)** average spectral transmittance curve after SNV.

Due to the detection limitations of the camera, there was a certain amount of instrument noise at the end of the original spectral curve. The spectral information of 336 bands in the 400-1000 nm wavelength range was selected as effective information. Then, the preprocessing of spectral data was completed with SG, MSC, and SNV, respectively. As shown in **Figure 5**, the spectral curve after SG filtering was relatively smooth as spectral noise was removed and the signal-to-noise ratio was improved. The phenomenon of baseline shift and offset was essentially eliminated after MSC treatment. In addition, SNV effectively eliminated the error caused by scattering.

The Kennard Stone (KS) algorithm was used to divide the dataset. The training set and the testing set had a ratio of 4:1. The spectral data preprocessed by four different methods were used as input variables to establish the SVM classification model. The classification accuracy of the model established by different pretreatment data was compared and tabulated in **Table 2**.

**Table 2:** SVM classification results of spectral data after different pretreatment.

Pretreatments	Training set	Testing set
None	80.8%	79.2%
SG	82.9%	80.0%
MSC	85.2%	81.7%
SVN	86.7%	80.8%

Compared with the model established from the original data, the accuracy of the model established from the spectral data processed by the three preprocessing methods was improved in both the training and test sets. In contrast, the classification accuracy of the spectral data model processed by SNV had the most significant increases at 5.9% and 1.6%, respectively. Therefore, SNV was used in subsequent model building to preprocess spectral data.

### 3.4 Feature extraction

**Figure 6:**
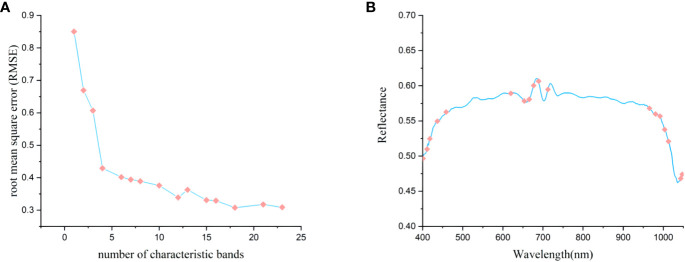
Characteristic bands selected by SPA. **(A)** the process of optimization and selection of characteristic bands; **(B)** the selection of 16 characteristic bands.

We used the SPA algorithm to extract characteristic bands from the original spectral data that were processed by the SNV algorithm. In this process, the root mean square error (RMSE) of the model was calculated based on the characteristic bands preselected at each iteration. The high accuracy of the model was based on the lowest RMSE. As shown in **Figure 6A**, the RMSE is roughly negatively correlated with the number of selected bands; when the number of characteristic bands was 16, the RMSE achieved its lowest value of 0.6628.

**Figure 6B** depicts the distribution of these 16 characteristic spectral wavelengths across the full spectral band. The selected characteristic wavelength bands were mainly concentrated around 450nm, 700nm, and 950nm, and a few characteristic wavelengths were selected at the remaining peaks and valleys. The main reason for this was that the feature selection process of the SPA algorithm was an unsupervised process, which was only analyzed from the distribution of independent variables. In addition, the wavelengths at 401.0, 404.4, 411.1, 412.8, 417.8, 422.9, 433.0, 527.0, 649.6, and 733.7 nm demonstrated that degradation degrees of *Pleurotus geesteranus* strains could be to a certain extent differentiated in the visible range. The wavelengths at 949.7 nm and 962.9 nm were mainly related to the O-H stretching (second overtone) in water and the CH and CH_2_ stretching (third overtone) in fat. The wavelengths near 980 nm (985.7, 993.3 nm) were assigned to the second overtone NH stretching in protein (Yu et al., 2018). The wavelength at 980 nm was due to O–H stretching the second and first overtones (Wu and Sun, 2013).

**Figure 7:**
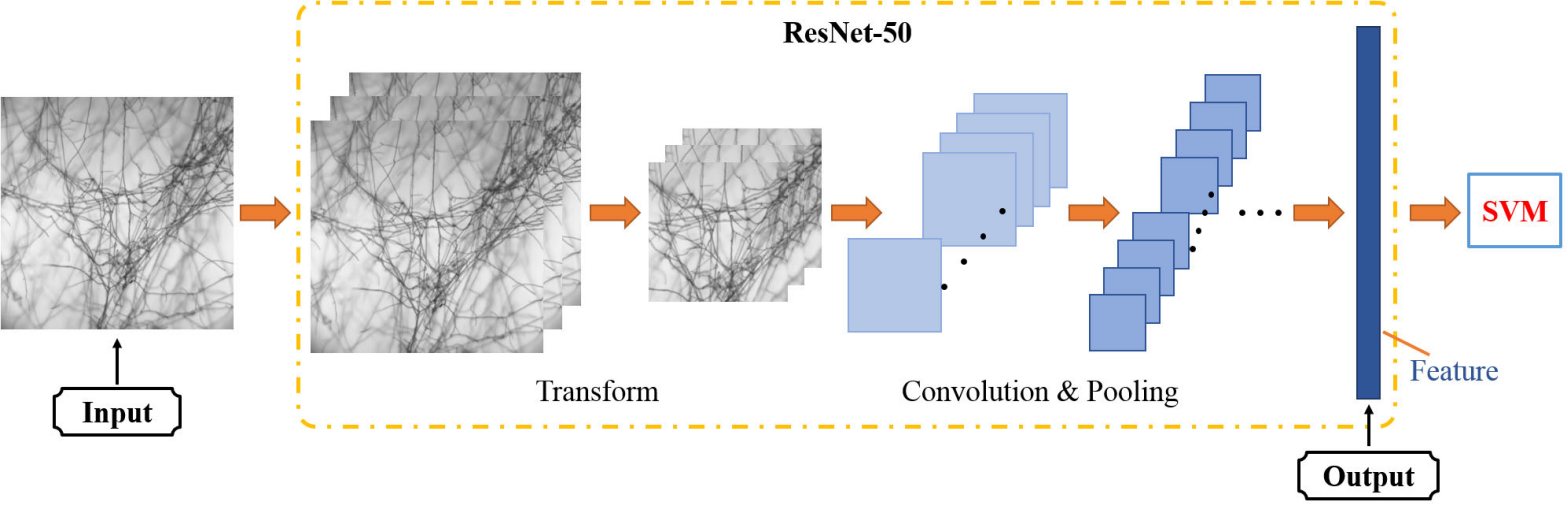
The structure of ResNet50 extracted feature model.

The grayscale image at the characteristic wavelengths of each micro-hyperspectral image was selected as the input. The pre-trained ResNet50 model was used to extract the features of each image. Since the pre-trained ResNet50 model used the ImageFolder function to read the image, the function copied the single-channel image automatically and converted it to a three-channel image at the same time. After converting each grayscale image into the RGB format, it reduced the image from the center to a size of 224 * 224, i.e., the input image size specified by the ResNet50 structure through the resize function. At the end of the process, the fully connected layer compressed the features extracted after convolution and pooling. It processed and integrated the output features of the previous layer into a 2048-dimensional feature vector through different weights. Then, the features were sent to the SVM classifier as the input. The model structure is shown in **Figure 7**.

### 3.5 Classification model

**Figure 8:**
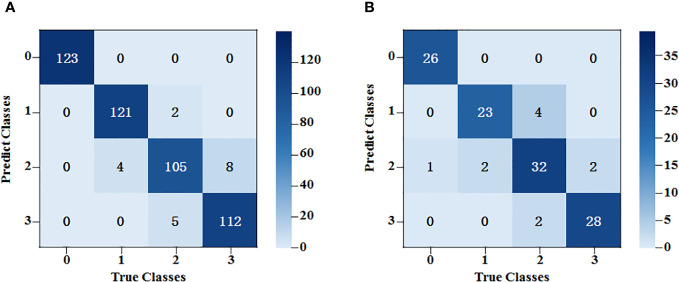
SVM classification results of the average spectral data at the characteristic wavelength. **(A)** The training set results of SPA-SVM; **(B)** The testing set results of SPA-SVM.

An SVM classification model was established for the spectral data based on characteristic bands after SNV. The radial basis (REF) function was chosen as the kernel function, and the criss-cross method was used to select the optimal parameters during modeling. As the classification results show in **Figure 8**, after the spectral data underwent pretreatment and feature extraction, the modeling accuracy was 91.0% for the training set and 83.3% for the testing set.

The accuracy of the model based on the feature wavelengths extracted by the SNV-SPA algorithm in the testing set was 4.1% higher than that of the original-spectra-input model. This difference indicated that the SNV-SPA algorithm could reduce the complexity of the model while improving its prediction ability. Therefore, it has better performance.

The features extracted from the depth residual model ResNet50 were used as the input of the SVM classifier, and the training accuracy was 87.7% and 80.8%. We also compared the results with the SVM model using all the grayscale images covering 336 bands as the input. With the “RBF” kernel function, the classification model accuracy was 80.2% in the training set and 71.1% in the testing set. The prediction accuracy of the ResNet50-SVM model was improved by 9.7% over the non-SVM model.

**Figure 9:**
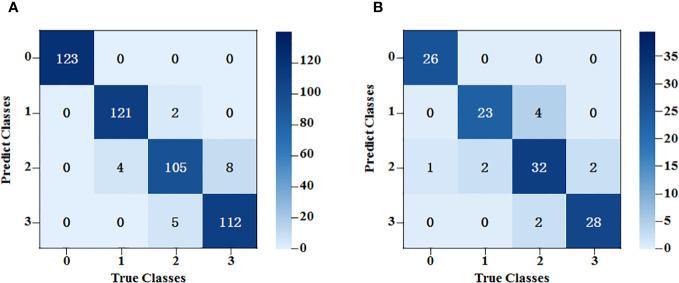
SVM classification results based on fusion of spectral data features and image data features. **(A)** The training set results of SPA+ResNet50-SVM; **(B)** The testing set results of SPA+ResNet50-SVM.

After feature extraction, the training kernel matrix and the testing kernel matrix were calculated based on the feature vectors under the spectral dimension and image dimension, respectively. All the results of SVM training and classification for each combination of fusion coefficients were compared using a grid search. After that, linear fusion was performed for the kernel matrix of the two different dimensions under the optimal weight. The final training results are shown in **Figure 9**. The accuracy rates of the training and testing sets were 96.0% and 90.8%, respectively.

Generally, the classification errors were mainly concentrated between Class 2 and Class 3. This might be because the spectral trends of these two classes were very similar and the shapes of the peaks and valleys had little difference, whereas, the peaks and valleys of Class 0 and Class 1 were quite different from them. In terms of image dimension, some mycelia of Class 2 and Class 3 were too narrow to be distinguished. By contrast, the mycelia of Class 0 and Class 1 could be detected easier because they were stronger.

To evaluate the model’s classification capability, each model was trained by SVM. The experimental results of the training and testing datasets are shown in **Table 3**. For spectral dimension data and image dimension data, it was found that the testing set accuracies of the models established after feature extraction using SG-SPA and ResNet50 algorithms were 4.1% and 9.7% higher than those of the original models, respectively.

**Table 3:** Comparison of classification results of SVM detection.

Data	Methods	Accuracy	
		Training set	Testing set
Spectrum	None-SVM	80.8%	79.2%
	SG-SPA-SVM	91.0%	83.3%
Image	None-SVM	80.2%	71.1%
	ResNet50-SVM	87.7%	80.8%
Spectrum + image	SPA+ResNet50-SVM	96.0%	90.8%

## 4 Discussion

From the discriminate results, it can be seen that the pre-processing and feature extraction methods might have had significant effects on improving the accuracy of classification models. The core of analyzing the image data features was to extract the external information of objects based on image information, such as color and texture. ResNet50 showed effectively performance in the image feature information extraction. In addition, the original grayscale image has problems such as mycelium occlusion and an unclear background, which could lead to a strong correlation between different image data. Therefore, the over-fitting phenomenon may occur if directly used in the training model. The ResNet50 effectively reduces the correlation of the original data, which could not only solve the over-fitting problem in the model but also improve its training speed.

In contrast to image information, spectral information mainly used the relevant characteristic wavelength of the internal material content of *Pleurotus geesteranus*. It was also noted that only using feature fusion modeling is better than spectral feature modeling or image feature modeling with image feature modeling. These findings imply that feature fusion combines feature information from different types of sources, which reduces the dependence of the model on a single feature to a certain extent and increases the interpretability of features. Thus, the reliability of information would be maintained, while the accuracy and robustness of the model would be simultaneously improved. To sum up, compared with the traditional method of manually identifying strain degradation, hyperspectral technology could more effectively and quickly identify whether the strain was degraded and its degree of degradation.

## 5 Conclusion

This study compared different pre-treatments and models to establish the methods for non-destructive detection of strain degradation in *Pleurotus geesteranus* using micro-hyperspectral imaging. Texture features were extracted from the images under the 16 feature bands that were chosen by the SPA algorithm. The results show that the SVM model established after feature extraction is optimal, with a classification detection testing set accuracy of 90.8%. Based on the above findings, using the micro-hyperspectral image information of the strains to establish a detection model could be a rapid and non-destructive approach to identifying the degradation of *Pleurotus geesteranus* strains. This approach was demonstrated to have high detection speed and accuracy. It may provide new insights and methods for the early identification of edible fungi strain degradation and the acquisition of edible fungi phenotype information. Furthermore, it will have good development prospects in the field of biological information breeding technology.

## Data availability statement

The raw data supporting the conclusions of this article will be made available by the authors, without undue reservation.

## Author contributions

XW: Conceptualization, Writing – original draft, Funding acquisition, Writing – review & editing. SL: Investigation, Methodology, Writing – original draft. CX: Investigation, Methodology, Writing – original draft. WF: Formal Analysis, Writing – review & editing. CD: Software, Writing – review & editing. ZW: Resources, Supervision, Writing – review & editing. DY: Resources, Methodology, Writing – review & editing. DJ: Conceptualization, Project administration, Writing – review & editing.
